# Primary palliative Care in General Practice – study protocol of a three-stage mixed-methods organizational health services research study

**DOI:** 10.1186/s12904-018-0276-6

**Published:** 2018-01-30

**Authors:** Helen Ewertowski, Fabian Tetzlaff, Stephanie Stiel, Nils Schneider, Saskia Jünger

**Affiliations:** 1 Institute for General Practice, Hannover Medical School, Carl-Neuberg-Straße 1, 30625 Hanover, Germany; 2 Cologne Center for Ethics, Rights, Economics and Social Sciences of Health, University of Cologne, Universitätsstraße 91, 50931 Cologne, Germany

**Keywords:** Primary palliative care, General practice, Organizational health services research, Complex intervention

## Abstract

**Background:**

The focus of this project is on improving the provision of primary palliative care (PC) by general practitioners (GPs). While approximately 10–15% of the incurable, seriously ill or dying people will be in need of specialist PC, the vast majority can be adequately treated within generalist care. The strengthening of the GP’s role in PC, as well as ensuring close collaboration between specialist PC services and GPs have been identified as top priorities for the improvement of PC in Germany. Despite healthcare policy actions, diverse obstacles still exist to successful implementation of primary PC on a structural, process, and economic level. Therefore, this project aims at addressing barriers and facilitators to primary PC delivery in general practice in Germany.

**Methods:**

The study follows a three-step approach; first, it aims at systematically analyzing barriers and facilitators to primary PC provision by GPs. Second, based on these outcomes, a tailored intervention package will be developed to enhance the provision of primary PC by GPs. Third, the intervention package will be implemented and evaluated in practice. The expected outcome will be an evidence-based model for successful implementation of primary PC delivery tailored to the German healthcare system, followed by a strategic action plan on how to improve current practice both on a local level and nationally.

**Discussion:**

The first step of the project has been partly completed at the time of writing. The chosen methodologies of four sub-projects within this first step have opened up different advantages and disadvantages for the data collection. In sum of all sub-projects, the different methodologies and target groups contributed valuable information to the systematic analysis of barriers and facilitators to primary PC provision by GPs.

**Trial registration:**

The study (BMBF-FK 01 GY 1610) was retrospectively registered at the German Clinical Trials Register (Deutsches Register Klinischer Studien) (Registration N° DRKS00011821; date of registration: December 04th 2017) and at the German Register of health care research (Versorgungsforschung Deutschland - Datenbank) (Registration N° VfD_ALLPRAX_16_003817; date of registration: March 30th 2017).

## Background

In Germany, as in many other high-income countries, it is anticipated that both the proportion and the actual numbers of people who should receive palliative care (PC) is increasing considerably [1–3]. The remit of PC has expanded since its relevance for other diseases than cancer has been widely acknowledged; in addition, integration of PC at an earlier stage in the disease process – not only close to death – has been shown to be beneficial [2]. In addition, the demand in primary care will rise with an ageing population and changing patterns of mortality – people at older ages often suffer from multiple chronic diseases of indeterminate prognosis [4]. According to estimates by the German Association for Palliative Medicine (DGP), up to 90% of the approximately 850,000 people who die in Germany each year will be in need of PC [3]. Of those, about 10% will require specialist PC at some point, while the majority will need primary PC. It is estimated that general practitioners (GPs) on average care for 3–4 patients with PC needs per quarter; however, these figures presumably underestimate the real care extent since these estimates do not appropriately reflect care for patients with non-oncological conditions [5]. The situation of patients in need of PC in a general practice is characterized by multi-morbid patients with a broad spectrum of conditions and a predominance of non-oncological chronic diseases such as cardiovascular or pulmonary diseases [6]. From a public health perspective, these trends demand for new approaches to service delivery; research into models for successful implementation of primary PC is therefore paramount.

### International evidence base regarding primary palliative care

Primary PC can be defined as “the clinical management and care coordination including assessment, triage, and referral using a palliative approach for patients with uncomplicated needs associated with a life limiting illness and/or end of life care. Has formal links with a specialist palliative care provider for purposes of referral, consultation and access to specialist care as necessary” [7]. Evidence from international research consistently underlines the importance of PC in a generalist setting as an indispensable element in the continuum of care provision, as well as the pivotal role of the GP in the provision of primary PC [8, 9]. In a number of countries, the fundamental right for patients of all diagnoses to receive PC from an early point in the course of a life-limiting condition is now anchored in policy and legislation, involving an increased emphasis upon the role of primary PC [2]. It has been argued that a wise combination of generalist and specialist PC constitutes a more sustainable and cost-effective approach to care provision [10]. Accordingly, in the past decade, important scientific, policy and advocacy initiatives were developed internationally to facilitate primary PC provision by GPs and to support their endeavors [11–14].

### Challenges related to the provision of primary palliative care in general practice

Despite the widely acknowledged importance of GPs’ engagement in primary PC [11], a number of - partly interrelated - challenges were identified related to the delivery of primary PC in practice; these concern 1) structural barriers (e.g. GP undersupply and uneven distribution in rural versus urban areas), knowledge barriers (e.g. lack of knowledge, skills, and clinical routine in providing PC; practical obstacles to undertake training), 2) service barriers (e.g. lack of active GP involvement in transition from curative to PC), and 3) consequences of the “specialization” of PC (e.g. unclear role definitions and lack of clarity regarding roles and responsibilities of generalist PC and communication and collaboration between generalist and specialist care providers) [2, 15].

### German context

Position papers and professional guidelines by leading authorities [16, 17], as well as nationwide consented public health actions to improve PC provision in Germany [18], define generalist PC as the foundation of all PC concepts and emphasize the pivotal role of the GP in PC delivery. Research confirmed that GPs in Germany regard it as their responsibility and as part of their professional self-perception to care for their patients throughout the whole disease trajectory, including provision of end-of-life care. The crucial importance of strengthening PC by GPs was even anchored in the ‘hospice and palliative care law’ passed in 2015 [19]. However, to date it has not been clearly defined what PC in general practice exactly entails [20]. The clinical guideline for PC for cancer patients [16] details generalist PC as follows: palliative basis and follow-up assessment of the patient’s status; the treatment of symptoms of low to medium complexity; involvement of specialist PC if appropriate; and identification of treatment goals in agreement with the patient. The ideal situation described in the law and in the clinical guideline is, however, far from being implemented into practice, and a pronounced undersupply of patients with generalist PC has been stated [3]. Routine data of health insurances indicate that primary PC occurs too late in the course of a life-limiting disease, and predominantly for patients with oncological diseases, confirming the still prevailing idea among GPs that PC is primarily applicable close to a patient’s death and in the context of cancer care [3]. Until now, the regulatory framework for primary PC delivered by GPs merely consists of four specific billing codes introduced in the medical compensation system in 2013, while not allowing for statements regarding the quality of the care provided. In addition, the codes only refer to single services by GPs, while nursing and other care is not included. This is critically discussed since these billing codes do not ensure essential features of holistic PC provision such as coordination, network structures, and multi-professional care. Particularly the team approach, a key element of successful and sustainable PC provision, is not adequately considered in the current regulations for primary care. Unlike with specialist palliative home care, where conditions, admission criteria, and care activities have been defined, for generalist palliative care this is very vague, bearing confusion and uncertainties for professionals, patients and families, as well as other involved stakeholders [20]. The intent to strengthen primary PC economically by improving remuneration for services delivered by primary care providers was recently anchored in law [19]. However, until now, no systematic approaches for the practical implementation of these innovations are in place, and empirical evidence on costs and benefits of respective care models is lacking.

GPs providing PC in Germany are confronted with the difficulty of integrating the demands of end of life care with a generalist caseload that usually implies many competing priorities [2, 21, 22]. In international comparison, physicians in Germany showed the highest self-assessed workload and the greatest number of patient contacts, with at the same time the shortest time per patient contact of only 9.1 min [23]. There is a tension between a pressurized context of care [2] and the nature of PC work with its unpredictable demands, uncertainty of illness trajectory, and its difficulty in planning, its intensiveness, and its personal strains [21]. Interactions with patients in the context of PC provision tend to require high investment of time, communicative and emotional effort, and tolerance towards uncertain outcomes. These requirements can become incompatible with the other demands on a GP in his or her every day practice [24]. For example, frequent and time-consuming home visits may be difficult to cope with in a regular every day practice characterized by high workload and time pressure. In addition, depending on the regional or local care infrastructure, GPs often feel as “lone fighters” in providing PC for their patients, while team composition and access to specialist support were emphasized as crucial for the capacity to provide PC in a generalist setting [22].

In summary, a first foundation of research [6, 24], education and professional guidance [16, 25], and policy initiatives [17, 19] has been laid for enhancing primary PC delivery in Germany. However, the scientific evidence base and the conceptual development are not as far advanced as in countries such as the UK, the Netherlands, or Australia. To date, a comprehensive program for enhancing primary PC has not been developed. Although core findings and recommendations from other countries may be partly transferrable, the concrete practice largely depends on the particular conditions of the national healthcare system and structural prerequisites in the regional context of healthcare provision, with implications for the role and responsibility of the GP in the provision of care.

## Aim and research question

This research project aims at providing an empirical basis for the sustainable implementation of primary PC by GPs. Sub-goals are:

- the systematic analysis of barriers and facilitators to primary PC provision using a theoretical framework from implementation science,
- the development of a tailored intervention package to enhance primary PC provision and support care providers to integrate the new processes with their everyday practice, and
- the implementation and evaluation of the intervention package to assess its practical applicability and its effects on patient care.

### Research questions

I. Analysis and operationalization of barriers to primary PC provision by GPs in the German healthcare context on a structural, educational, process, and economic level: Which mechanisms and formal regulations impede routine embedding of PC provision in GPs everyday clinical practice at individual and collective level?
II. Identification and operationalization of concrete measurable actions to improve the implementation of primary PC delivery by GPs: Which measures will have the potential to facilitate integration of end of life care with a generalist caseload and to enable the GP and his practice team to provide high quality PC to their patients? Which measures will be suitable to promote collaboration with other care providers relevant for PC delivery?
III. Testing and evaluation of a tailored intervention package for the facilitation of primary PC delivery by GPs in Germany: What is its practicality, as well as its impact on care and patient-relevant outcomes? How well does it score compared to other models of GP involvement in PC delivery?

## Methods and design

**Fig. 1:**
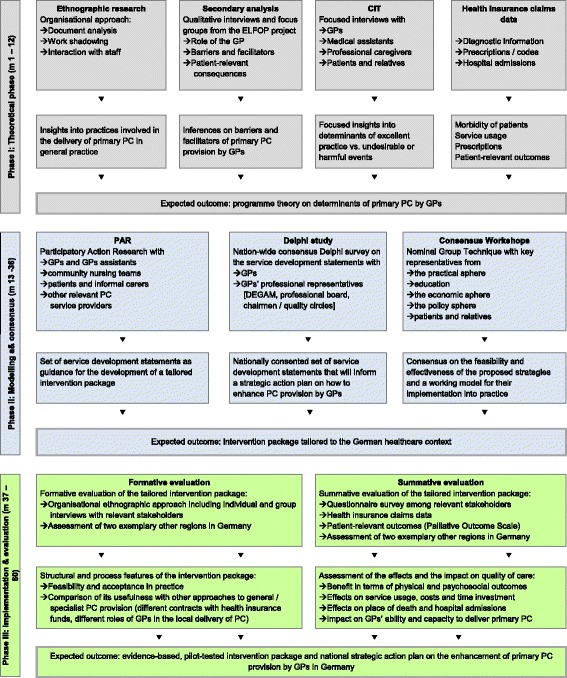
Design of the research project, modelled after Campbell et al. [26, 27]

To ensure scientific quality and rigor, the study design was developed in line with the recommendations for organizational health services research and theoretical foundation of health services research by the German Network for Health Services Research [26, 27]. The study encompasses three phases modelled after the framework for the development and evaluation of tailored interventions by Campbell et al. [26] (Fig. 1).

- The *theoretical phase* of this research is designed to gain an in-depth understanding of the mechanisms that determine the (un)successful delivery of primary PC in general practice.
- The *modelling phase* uses participatory methods and consensus building techniques in order to ensure ownership of the resulting intervention package and hereby enhance advocacy, dissemination, and the likelihood that the intervention will be successfully implemented into practice on the long term.
- The *implementation and evaluation phase* is designed to test and evaluate the acceptance, feasibility, and applicability of the intervention package in practice. Using formative evaluation, it will be examined how the individuals and groups involved in the delivery of primary PC engage in the mobilization of resources to secure consent, trust, and cooperation, as well as in the realization of activities that may lead to routine incorporation of elements of practice into everyday work. Summative evaluation will be employed to assess the outcomes in terms of the quality of patient care, the costs and benefits for the involved stakeholders, and the impact of the intervention package for the healthcare system.

### Study population and data collection

***Phase I – Theoretical Phase:*** The theoretical phase comprises four sub-projects.

First, an *organizational ethnographic field approach* will be applied to gain insights into the practices involved in delivering primary PC and to analyze barriers and facilitators. Data will be collected by means of document analysis (such as care plans, case notes, registers); by work shadowing (observation of daily routines, team meetings, staff interactions, home visits); and by interaction with staff (informal interviews, cognitive interviewing / think-aloud technique) [28]. Field notes will be taken according to a pre-defined scheme [29] and will be transformed into accounts including information about procedures, observations of actions and interactions, quotations of conversations, interpretations (e.g. of underlying attitudes), and the researcher’s intuitive thoughts and memos. These accounts will be imported into data files using software for qualitative text analysis (MaxQDA®).

*Second, a secondary analysis of qualitative data* [30] will be undertaken with a particular focus on barriers and facilitators of PC provision by GPs. Data are available from a recently concluded project on end of life care for frail older patients in family practice (ELFOP), a longitudinal study on needs, appropriateness and utilization of services (funding number: BMBF-FK 01 GY 1120) funded by the Federal Ministry of Education and Research. Participants were selected through theoretical sampling to include GPs with different backgrounds, working conditions (single or joint practice), and working in rural and urban areas characterized by diverse care infrastructures. Both the interviews and the group discussion include relevant information on the GPs’ perception of tasks, problems, challenges in the end-of-life care for frail older people, as well as on their perceived roles, responsibilities, and professional self-perception within this context of care.

Third, based on the findings from ethnographic research and secondary interview analysis focused interviews will be conducted with GPs, GP assistants or nurses from home care teams or inpatient service provider, patients, and their relatives (Table 1) using the *Critical Incident Technique* (CIT) [31, 32] to identify significant mechanisms that result in excellent practice versus in undesirable or even harmful events.

**Table 1:** Target/ study population detailed for each work package of data collection

Work package	Target population	Proposed sample size	Sampling strategy
Ethnographic research	GPs; GPs assistants, professional caregivers	*n* = 30	The Institute of General Practice’s education and research network of *n* = 250 general practices; theoretical sampling contrasting: rural vs. urban; single vs. joint practice; specific palliative education vs. nonspecific palliative education
Secondary analysis	GPs, GPs assistants, professional caregivers	interviews: *n* = 52 group discussions: *n* = 3	Existing data set of interviews / one focus group with GPs conducted within the ELFOP project; all interviews and the focus group are included
CIT	GPs, GPs assistants, professional caregivers, patients and relatives	*n* = 16	Theoretical sampling contrasting: rural vs. urban; diagnosis; single vs. joint practice; men vs. women
Analysis of Health insurance claims data	GPs’ patients identified as being in need of PC	not applicable	Health insurance claims data: all AOKN and BARMAR insured persons in Lower Saxony with palliative billing codes
PAR	GPs, community nursing teams, patients and informal carers, other relevant PC providers	not applicable	Theory-based purposive sampling [55]
Delphi study	GPs’ professional representatives and other relevant experts for health policy decision making and healthcare planning	*n* = 30	DEGAM; DGP; GPs’ chairmen / quality circles; the Institute of General Practice’s close links with GPs’ professional boards and associations (Hausärzteverband)
Consensus workshops	GPs’ professional representatives; health insurers; experts for medical education; and other relevant experts for health policy decision making and healthcare planning	*n* = 20	Two groups with approximately 10 participants each; theory-based purposive sampling [55]
Implementation of the intervention package	GPs’, community nursing teams, health insurers; and other relevant experts for health policy decision making and healthcare planning	not applicable	Exemplary test region in Germany (Lower Saxony) involving n = 8 general practices, as well as all relevant service providers and stakeholders in the respective region
Questionnaire survey (Summative evaluation)	GPs’, community nursing teams, health insurers; and other relevant experts for health policy decision making and healthcare planning	*n* = 130	Service providers, insurers, and other local stakeholders involved in the implementation of the tailored intervention package (Phase II) and from two exemplary other regions in Germany
Patient-relevant outcomes (Summative evaluation)	Patients and informal carers	standardized scales *n* = 32 interviews *n* = 16	Patients and informal carers attended by general practices who are involved in the implementation of the tailored intervention package (Phase II) and from two exemplary other regions in Germany

Fourth, *secondary analysis of health insurance claims data* of the AOK – Die Gesundheitskasse für Niedersachsen (AOKN, statutory health insurance) and BARMER (statutory health insurance), and from the GPs’ office software will serve to describe the morbidity and service usage of patients, assess patient-relevant outcomes such as hospital admissions, estimate the time investment and costs for the provision of primary PC by GPs, and to draw inferences on the relevant laws, professional regulations, and reimbursement systems (see Table 2).

**Table 2:** Outcomes, comparators, and data sources

Outcome	Measure(s), indicator(s)	Data source
Patient-related outcomes		
Quality of life	Physical and psychosocial wellbeing, social support, financial strain	Standardized quality of health / care related measures such as POS^a^; QODD^b^; QUELC^c^
Quality of care	Unplanned hospital admissions	Health insurance claims data
Service usage and time investment		
Service usage	GPs’ consultations and home visits; prescriptions (medication, medical aids, interventions); referral to specialists and other service providers; out-of-hours contacts; emergency interventions	Health insurance claims data GP’s office software
GPs’ time investment	Time dedicated to assessments, home visits, telephone consultations, physical care, and psychosocial care	Time registration (ethnographic field research)
Impact on GPs’ and practice staff’s ability and capacity to deliver primary palliative care		
Quality of palliative care delivery	GPs’ and practice staff’s appraisal of the quality of PC provided to their patients	Organizational ethnographic field research including individual and group interviews
Quality of care other than palliative care	GPs’ and practice staff’s appraisal of the quality of care provided to other patients	Organizational ethnographic field research including individual and group interviews
Work satisfaction	GPs’ and practice staff’s work-related satisfaction and sense of meaningfulness	Organizational ethnographic field research including individual and group interviews
Economic evaluation		
Direct costs for the healthcare system	Service usage (consultations and home visits, prescriptions of medicines and medical aids, specialist referrals, social services, out-of-hours contacts, emergency interventions)	Health insurance claims data GP’s office software
Direct costs for patients and relatives	Additional costs for medications and therapeutic interventions not covered by the health insurance funds	Organizational ethnographic field research including individual and group interviews
Indirect costs for patients and relatives	Social isolation; (temporary) work loss; abandon of activities or relationships	Organizational ethnographic field research including individual and group interviews
Direct costs for GPs and other healthcare providers	Costs invested for PC not remunerated within the medical compensation system	Organizational ethnographic field research including individual and group interviews
Indirect costs for GPs and other healthcare providers	Personal strain; impact on healthcare professionals’ private lives; abandon of activities or relationships	Organizational ethnographic field research including individual and group interviews
Feasibility of the intervention package		
Process monitoring and formative evaluation of the implementation phase	Normalization Process Theory core constructs (coherence, cognitive participation, collective action, and reflexive monitoring)	NoMAD^d^ assessment instrument for the evaluation of the implementation of complex interventions

^a^Palliative Outcome Scale [38]

^b^Quality of Dying and Death questionnaire [47]

^c^Quality of End of Life Care Questionnaire [55]

^d^Measure Development Based on Normalization Process Theory [56]

***Phase II – Modelling Phase:*** The modelling phase consists of three sub-projects building up on each other.

First, *Participatory Action Research* (PAR) [33] will be undertaken with general practices, community nursing teams, patients and informal carers, as well as other relevant PC service providers to define adequate operationalization of PC processes and quality outcomes, and to condense a set of service development statements [34, 35].

Second, a *nation-wide consensus Delphi study* will be undertaken with GPs and their professional representatives such as the GPs’ professional federation, the Medical Chamber, and the German association of general practice (DEGAM) to seek expert consensus on these service development statements.

Third, *consensus workshops* will be conducted in the follow-up of the *Delphi study*, using the *Nominal Group Technique* [36] to elaborate a strategic action plan on how to put the service development statements into practice. Workshop participants will be key representatives from the practical, education, economic, and policy sphere, as well as representatives of patients and relatives. Data collected during this phase will be integrated during analysis (see [2, 37]) to design the tailored intervention package.

Finally, an intervention package will be developed for enhancing the integration of PC provision into general practice, drawing on the example of the Gold Standards Framework for Palliative Care [12], but adapting it to the German context. A participatory framework and consensus building techniques will be applied to promote ownership of the developed elements and to enhance commitment among the relevant stakeholders.

***Phase III – Implementation and evaluation:*** The intervention package developed in phase II will be implemented and evaluated in an exemplary test region in Lower Saxony.

First, *formative evaluation* will use an organizational ethnographic approach including individual and group interviews with relevant stakeholders to (a) test the feasibility and acceptance of the intervention in practice, and (b) to compare the intervention to two exemplary other regions in Germany with different approaches to generalist and specialist PC provision in terms of contracts with health insurance funds, different roles of GPs in the local delivery of PC (e.g. Westphalia-Lippe).

Second, *summative evaluation* will be accomplished to assess the effects of the intervention on patient care in terms of physical, practical, and psychological outcomes. Data sources will be a questionnaire survey among the relevant stakeholders involved in PC provision; analysis of clinical and health insurance funds routine data to assess the effects on patient care, service usage, and costs of care; and perceptions of patients and relatives regarding the outcomes of care using established instruments such as the Palliative Outcome Scale [38] (Table 2).

### Sample size calculation

Phase I: For the ethnographic field research the participation of *N* = 8 GPs, GP assistants and other medical professionals was planned considering methodological arguments of theoretical sampling when contrasting practices characteristics such as rural vs. urban, single vs. joint practice, men vs. women. At least *N* = 16 interviews following the Critical Incident Technique were proposed which relates to the methodological experience that data saturation in qualitative analysis via Grounded Theory. Data collection will end once data saturation is obtained which will be the case when no more new or valuable information can be attained, further coding is no longer feasible, and once the study can be replicated [39]. This usually takes place after 12–15 interviews. The sample size of secondary analysis of qualitative data from ELFOP is pre-determined due to the retrospective design of the sub project. The sample size of the secondary analysis of health insurance claims data is not applicable and will be defined by the amount of specific data sets of the current insured persons of the AOKN and BARMER.

Phase II: Reflecting the current literature on successful Delphi studies, *N* = 30 participants who complete each Delphi round are planned. Due to the expected drop-out of 60% during the course of the Delphi assessment, probably three times as many participants will be initially invited to this sub project [18]. The final consensus workshops aims to include *N* = 20 stakeholders following a theory-based purposive sampling [40].

Phase III: For the questionnaire survey in terms of the summative evaluation a participation of *N* = 130 key stakeholders of local (primary) palliative care delivery from the practical, education, economic, and policy sphere, as well as representatives of patients and relatives is proposed based on content-related reflections taking the accumulated sample size of the prior subprojects into account.

### Methods of data analysis

***Phase I + III:*** *Analysis of qualitative data* (ethnographic field research, CIT, secondary analysis of interviews and focus group), as well as for the formative evaluation during the *implementation phase* (organizational ethnographic approach with individual and group interviews) will be guided by the methodology of Grounded Theory. For each qualitative data-subset (field notes, interviews, focus group), Strauss and Corbin’s single coding paradigm [41] will be used to identify salient determinants and mechanisms of relevance to primary PC delivery in general practice. To ensure inter-subjective validity and reliability, qualitative data will be analyzed independently by the members of the junior research group and discussed within the team until consensus on core categories will be reached. Analysis of health insurance claims data and GPs’ office software will follow the guidelines for best practice of secondary data analysis [42]. For quantitative data analysis univariate and bivariate descriptive methods as well multivariate methods (e.g. logistic regression or survival analysis [43, 44]) of empirical social research will be used. The findings from the different data sources within the *theoretical phase* will be triangulated to draw inferences on the structural, economic, and interactional mechanisms that either hamper or facilitate PC delivery in general practice.

***Phase II:*** A characteristic feature of *Participatory Action Research* is community and practice partners’ participation also in the phase of data analysis [45]. Mutual reflection, shared learning and co-construction of meaningful outcomes are key elements of this process; for this aim, pragmatic and comprehensible methods are recommendable such as thematic or content analysis, or the single coding paradigm. A stepwise approach to data analysis will be conducted in working groups composed of academic staff and representatives from the field [45]. Outcomes of the *Nominal Group Technique employed during the consensus workshops* will be analyzed using quantitative (descriptive comparison between ranks and weights of ideas and statements) and qualitative methods (combination of structured-thematic and formal qualitative content analysis) [46].

The Delphi study and the questionnaire survey will be analyzed using descriptive statistics (percentages, measure of central tendencies such as mean and median values, and measures of dispersion such as variance, interquartile ranges, and standard deviation). For the Delphi study, results of concluded survey rounds will be processed to provide feedback to the participants in the subsequent round [47]. If necessary, service development statements will be modified based on the experts’ responses in order to inform the questionnaire for the next survey round and hereby improve the degree of consensus within the expert panel. For the development of the tailored intervention package, data from the different sources in the modelling phase will be integrated in accordance with the framework for the design of complex interventions [26] and the corresponding guidance for synthesizing and triangulating mixed methods data (48). Following the recommended phases of the mixed methods analysis process [48], a package of expedient interventions will be proposed and elaborated based on the data collected during the participatory process, will be translated into service development statements, and consented during the subsequent Delphi survey and consensus workshops.

Statistical analysis will be compared and contrasted with qualitative data on direct and indirect costs resulting from organizational ethnographic research including individual and group interviews using triangulation. Standardized patient-related outcomes such as Palliative Outcome Scale [38] or Quality of Dying and Death questionnaire [49] (Table 2) will be analyzed according to the instructions for scoring, analysis, and interpretation of responses indicated by the authors in the instruments’ manual, on the respective website, or in key publications.

All qualitative analysis will be conducted using software for qualitative text analysis (MAXQDA®). Quantitative data will be analyzed using IBM SPSS Statistics (Version 24) and R (Version 3.3.0).

### Expected results

Following the principles of organizational health services research [26, 27] and implementation science [37, 50], significant results and knowledge gain of this project are expected at different stages of the research process and on different levels of changes in practice.

- The expected outcome of the *theoretical phase* will be a salient program theory [26] on determinants of primary PC in general practice. This will include specific knowledge on barriers impeding routine embedding of PC provision in general practice and its consequences for patient care.
- The *modelling phase* is expected to generate (a) a set of service development statements [34, 35] that will serve as guidance for the development of a tailored intervention package and that will be submitted to a consensus building process in order to substantiate feasibility and applicability of the proposed actions on a broader professional basis; and (b) an intervention package tailored to the German health care context that will enable GPs to provide PC to their patients according to their professional standards and to the best of the patient’s needs and requirements. The intervention package will be designed to address the barriers identified in phase one and will include flexible modules that can be adapted to different local or regional conditions. The insights gained during this phase will deliver specific information on measurable actions to improve the conditions for GPs to provide high quality primary PC.
- The expected outcome of the *implementation and evaluation phase* will be an evidence-based, pilot-tested intervention package and a national strategic action plan on the enhancement of primary PC delivery by GPs in Germany. This will comprise knowledge on its practicality, its impact on care and patient-relevant outcomes, as well as on suitable quality indicators for primary PC in general practice.

## Discussion

In the meantime, the theoretical phase I of the project is partly completed. In the first sub-project, the *organizational ethnographic field approach*, a total of ten GPs, eleven GPs’ assistants and nine professional caregivers were accompanied by a scientific co-worker of the research team in their daily work. The method of data collection was proven very suitable for answering the research question. It allowed observing and understanding the broad spectrum of GPs’ and their assistants’ daily work and gave valuable information on the routine of GPs’ practices and the specific demands and financial background of delivering palliative care for GPs. By gaining comprehensive insights it has become possible to draw conclusions on barriers and facilitators to primary PC provision.

Second, the *secondary analysis of qualitative data* comprised 52 longitudinal interviews with 14 GPs carried out at up to four time points in six-monthly intervals. Additionally, three focus groups with GPs (*n* = 5), medical assistants (*n* = 6), and professional caregivers (*n* = 11) were considered. Analyzing this extensive data material allowed investigating the GPs’ perspective on and actions in end-of-life care. The interviews particularly contributed to the understanding of GPs’ discrimination and definition of patients in need of geriatric or palliative care.

Third, focused interviews with each four GPs, GPs’ assistants or professional caregivers from palliative or nursing home care, patients, and relatives using the *CIT* [31, 32] were conducted. The method was very helpful to assess different perspectives of care provider and care recipients and to distinguish which care contexts are perceived successful and positive or inappropriate and negative. Nevertheless, for patients and relatives it was difficult to distinguish in their reporting between a positive and negative example and not to integrate and mix both positive and negative care events. This way of describing care situations was less complex for GPs and other care professionals.

The *secondary analysis of health insurance claims data* of the AOK – Die Gesundheitskasse für Niedersachsen (AOKN, statutory health insurance) and BARMER (statutory health insurance) foreseen for the fourth sub-project of phase I is still ongoing and will probably continue until the end of the second project year. Though good relations between the research team and the authorities of the health insurances were developed, the provision of data was delayed due to technical problems or heavy workload. The clarification of data protection and legal issues, the conclusion of contracts and data transfer took more time and required more resources of all engaged persons than considered in the study design during grant application.

Overall, this project has the potential to substantially contribute to the practical elaboration of enhanced approaches to primary PC service provision with evidence-based intervention models, in particular with respect to the enhancement of collaboration and networking. Significant benefits for patients and their relatives are expected from improved primary PC delivery and improved collaboration with PC specialists, such as earlier identification of patients in need of PC, better assessment and treatment of pain and other symptoms, advanced care planning, and avoidance of unwanted life-prolonging treatments. But also benefits for healthcare professionals can be expected.

### Ethical issues

The methodological approach of this project does not involve any direct interventions or exposures to patients. However, several elements of data collection, data analysis, and publication of results require careful attention. PC is a sensitive field of health care with particular challenges for research; severely ill and dying people are considered a particularly vulnerable group and data collection can be potentially burdensome for participants [51]. The legitimacy of involving patients and relatives in scientific research has therefore been subject to debate in terms of research ethics. Evidence from studies and systematic reviews on ethical issues of research in PC suggests that – a diligent and respectful approach provided – research is both ethically justifiable and also desirable [51, 52]. Based on this evidence, a conceptual framework for ethics and data protection was elaborated for this project, taking particular account of a reasonable balance between effort, costs, and potential burden for participants, and the expected knowledge gain. During data collection, the participants’ health condition will be considered when choosing methods and settings of inquiry; care will be taken to keep periods of inquiry short.

The nature of ethnographic field research and PAR on the one hand bears ethical challenges such as problems of attaining informed consent; the power of interpretation; or dealing with secrets [53]. On the other hand, this type of research offers the opportunity of respectful interaction with all involved actors [51]. Consent and trust are dynamic and interactive concepts, and care will be taken to continuously sustain these throughout the research process by ensuring mutuality and creating an “ethical space” between researchers and partners in the field [54]. Research activities will be conducted in line with international ethics guidelines for ethnographic studies [55] and PAR [45], hereby assuring compliance with the principles of beneficence, non-maleficence, and protecting the autonomy, wellbeing, safety, and dignity. Since it will not be possible to attain individual informed consent from all participants – including patients – communication and comprehension of information, and voluntary participation will be assured by creating the highest possible transparency, e.g. through posters and flyers in waiting rooms of general practices, or the researcher wearing a badge identifying him as such. Regular supervision will be provided for the researchers in the field. Ethical considerations regarding *secondary analysis of qualitative data* mainly include concerns about reusing data for a different purpose without having the explicit consent from participants [56]. This will not be applicable here since this study can be considered a follow-up of the original project; data collection referred to a similar context of practice, and the participating GPs consented that data may be used for scientific purposes in terms of developing an empirically grounded intervention that builds on the project’s findings. In ethical and economic terms of undertaking research, secondary analysis will support careful use of already invested funding and resources for data collection, and avoid unnecessary burden on participants [30]. A data protection concept will ensure anonymity of individual persons by systematic anonymization and pseudonymization of collected data. A final version of the study protocol including a data protection plan was submitted for approval to the ethics committee of Hannover Medical School.

### Dissemination and implementation

Engaging partners during the project in the process of implementation and dissemination of the research findings will be ensured by the research design that substantially builds on participatory and consensus building techniques. A key feature of these methods is the creation of ownership of the outcomes and hereby enhancing incorporation into practice and impact on healthcare. It is inherent to the principles of PAR that it involves participation from partners at all stages of the research process, including implementation and dissemination [45]. This will help identifying relevant audiences for the outcomes of this project, as well as suitable strategies and contexts for dissemination and implementation. Since the relevant actors regarding care practice, education, financing, laws and regulations, as well as policy decision making will be involved in the core developmental stages of the tailored intervention package, translation of the outcomes into targeted action will be promoted not only at the end of the project (“end of grant knowledge translation”), but already throughout its duration (“integrated knowledge translation”) [45]. Ongoing benefit for the practice of primary PC provision is expected by reciprocal learning and mutual elaboration of strategies for improving action throughout the collaboration within the project. The expected product of this project, i.e. an evidence-based, pilot-tested intervention package and a national strategic action plan on the enhancement of primary PC provision by GPs in Germany, will be disseminated via diverse channels to ensure that all relevant audiences will be reached. Next to publication in scientific journals and presentation at (inter)national conferences, the following dissemination formats will be envisaged: executive summaries for health policy decision makers and health insurance funds stating the need and recommendations for action to improve conditions for primary PC provision in general practice; tools for practice, developed and consented during the modelling phase and refined during the implementation phase of the project; press information and personal stories illustrating the impact of adequate primary PC provision for the media; and position papers for professional associations in general practice and PC including recommendations for practice and for curriculum development. Formats of presentation and dissemination will be elaborated in collaboration with the partners in the field to ensure connectivity for different target audiences. Dissemination will be supported by the research institute’s close links to health policy makers, professional boards and associations, health insurance funds, as well as national and international collaboration in PC and health services research.

## Abbreviations

AOKN: AOK – Die Gesundheitskasse für Niedersachsen
CIT: Critical Incident Technique
DEGAM: German Association of General Practice
DGP: German Association of Palliative Care
ELFOP: End of life care for frail older patients in family practice
GP: General practitioners
PAR: Participatory action research
PC: Palliative Care
